# Computer-Aided Lead Optimization: Improved Small-Molecule Inhibitor of the Zinc Endopeptidase of Botulinum Neurotoxin Serotype A

**DOI:** 10.1371/journal.pone.0000761

**Published:** 2007-08-22

**Authors:** Jing Tang, Jewn Giew Park, Charles B. Millard, James J. Schmidt, Yuan-Ping Pang

**Affiliations:** 1 Computer-Aided Molecular Design Laboratory, Mayo Clinic, Rochester, Minnesota, United States of America; 2 Division of Biochemistry, Walter Reed Army Institute of Research, Silver Spring, Maryland, United States of America; 3 Department of Cell Biology and Biochemistry, United States Army Medical Research Institute of Infectious Diseases, Frederick, Maryland, United States of America; Cairo University, Egypt

## Abstract

Optimization of a serotype-selective, small-molecule inhibitor of botulinum neurotoxin serotype A (BoNTA) endopeptidase is a formidable challenge because the enzyme-substrate interface is unusually large and the endopeptidase itself is a large, zinc-binding protein with a complex fold that is difficult to simulate computationally. We conducted multiple molecular dynamics simulations of the endopeptidase in complex with a previously described inhibitor (*K*_i_^app^ of 7±2.4 µM) using the cationic dummy atom approach. Based on our computational results, we hypothesized that introducing a hydroxyl group to the inhibitor could improve its potency. Synthesis and testing of the hydroxyl-containing analog as a BoNTA endopeptidase inhibitor showed a twofold improvement in inhibitory potency (*K*_i_^app^ of 3.8±0.8 µM) with a relatively small increase in molecular weight (16 Da). The results offer an improved template for further optimization of BoNTA endopeptidase inhibitors and demonstrate the effectiveness of the cationic dummy atom approach in the design and optimization of zinc protease inhibitors.

## Introduction

Botulinum neurotoxin serotype A (BoNTA) is a protein produced by the spore-forming anaerobic bacterium, *Clostridium botulinum*. The neurotoxin consists of a light chain (M_r_ ∼50,000) and a heavy chain (M_r_∼100,000) linked covalently by a disulfide bond. BoNTA poisoning selectively inhibits the release of acetylcholine from presynaptic nerve terminals at neuromuscular junctions, thus causing flaccid paralysis and leading to prolonged mechanical ventilation with serious medical sequelae or death following respiratory arrest [1]. In small doses BoNTA is an effective medical treatment for a variety of cholinergic nerve and muscle dysfunctions [2], [3]. In addition to its medical applications, BoNTA has gained notoriety as a bioterror agent [4]. Because there is no antidote to BoNTA, effective small-molecule inhibitors of BoNTA are highly sought after as antidotes and as potential medical tools for modulating clinical uses of the toxin.

**Figure 1:**
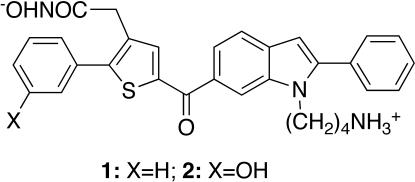
Chemical structures of inhibitors 1 and 2.

The light-chain domain of BoNTA is a zinc endopeptidase that specifically cleaves SNAP-25, a neuronal protein required for acetylcholine release [5]. The endopeptidase has been used as a target for developing small-molecule inhibitors of BoNTA [6]–[15]. Recently, we reported the development of a serotype-selective, small-molecule inhibitor of BoNTA with a *K*_i _of 12±2.6 µM (**1**, Figure 1) [7]. Developing or optimizing small-molecule inhibitors of BoNTA endopeptidase is, however, as challenging as developing small-molecule inhibitors of protein-protein complexes, a challenge that has been known for decades [16]. The challenge in the case of BoNTA endopeptidase inhibitors can be partly attributed to the substrate that wraps around the circumference of BoNTA endopeptidase upon binding and creates a substrate-enzyme interface of 4840 Å^2^ [17]. This large interface requires a high-affinity small molecule to block it. The extent of this challenge can be appreciated by comparing the interface area of BoNTA endopeptidase (4840 Å^2^) to the typical protein-protein interface area of 750–1500 Å^2^ [16]. Another part of the challenge in developing and optimizing these inhibitors is the endopeptidase itself, which is a large zinc-binding protein with a complex fold that is capable of large-scale conformational changes in solution and that is therefore difficult to simulate computationally [17]. Consequently, computer-aided optimization of BoNTA endopeptidase inhibitor leads has not been reported until now.

We report computer-aided optimization of **1** to arrive at an improved, serotype-selective, small-molecule BoNTA endopeptidase inhibitor with an “apparent” *K*_i_ (*K*_i_^app^) of 3.8±0.8 µM. This optimization was guided by multiple molecular dynamics simulations (MMDSs) of the zinc-containing endopeptidase in complex with **1** using the cationic dummy atom (CaDA) approach. This approach introduces four identical dummy atoms in a tetrahedral geometry around the zinc ion and transfers all the atomic charge of the zinc divalent cation evenly to the dummy atoms. The four peripheral atoms are “dummy” in that they interact with other atoms electrostatically but not sterically, thus mimicking the *4s4p^3^* vacant orbitals of the zinc divalent cation that accommodate the lone-pair electrons of zinc coordinates [18]–[23]. The results offer an improved template for further optimization of BoNTA endopeptidase inhibitors and demonstrate that the CaDA approach is useful for both design and optimization of zinc protease inhibitors.

## Results

### Design

Inhibitor **1** was designed to coordinate the zinc divalent cation embedded in the active site of BoNTA endopeptidase for affinity and simultaneously to interact with serotype-specific residues in the active site for selectivity [7]. This design was based on previous MMDSs using the CaDA approach. The MMDSs (20 simulations) of the endopeptidase in complex with **1** showed that (1) the hydroxamate group coordinated the active-site zinc ion; (2) the phenyl group substituted at the thiophene ring had a π-π interaction with Phe193 and a cation-π interaction with Arg362; (3) the indole ring was engaged in a cation-π interaction with Lys165; (4) the phenyl group attached to the indole ring had a van der Waals interaction with the side chain of Leu527 and a cation-π interaction with Lys165; (5) the ammonium group interacted with the carboxylates of Glu54 and Glu55 [7]. The absolute free energy binding between **1** and the endopeptidase was estimated to be −7.5 kcal/mol according to a free energy perturbation calculation of the MMDS-derived model of the **1**-bound endopeptidase using a published method [24] with modifications described in MATERIALS AND METHODS. These computational observations were consistent with the experimentally determined *K*_i_^app^ of 7±2.4 µM (ΔG ranging from –6.9 to –7.3 kcal/mol) for **1** (see below). In this context, the MMDS-derived model of the **1**-bound endopeptidase was used for the following lead optimization.

**Figure 2:**
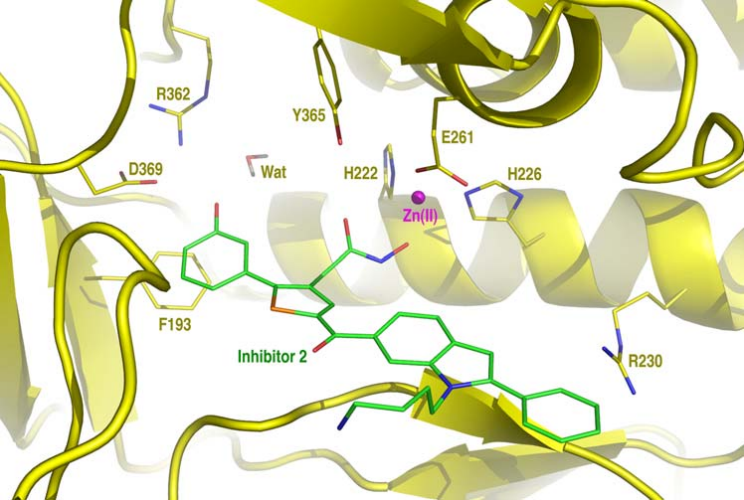
A close-up view of inhibitor 2 binding at the active site of the botulinum neurotoxin serotype A endopeptidase. The 3D model was generated by averaging 10,000 instantaneous structures obtained at 1.0-ps intervals during the last 0.5-ns period of 20 molecular dynamics simulations (2.0 ns for each simulation with a 1.0-fs time step and a unique seed for initial velocities) followed by 200 steps of energy minimization of the average structure of the entire complex.

An analysis of the MMDS trajectories of the endopeptidase in complex with **1** identified a vacancy around the *meta* position of the phenyl group substituted on the thiophene of **1**. Synthetically, this void can be filled by a hydroxyl group substituted at the phenyl ring. This hydroxyl group can form hydrogen bonds with active-side residues to improve the affinity for the endopeptidase and the introduction of this hydroxyl group can also increase the hydrophilicity of **1** because dimethyl sulfoxide is needed to dissolve **1** in water. These considerations led to the design of inhibitor **2** (Figure 1). MMDSs (20 simulations) of the endopeptidase in complex with **2** were carried out to confirm the anticipated hydrogen bonds. The result of these simulations suggested that **2** binds at the active site of BoNTA endopeptidase in a manner similar to that of **1** and that the hydroxyl group attached to the phenyl group of **2** indeed has hydrogen bonds with Arg362 and Asp369 of the endopeptidase (Figure 2). In the average structure of the endopeptidase complex obtained from 10,000 instantaneous structures at 1.0-ps intervals during the last 0.5-ns period of the 20 different simulations using an explicit water model [25], the hydrogen bond of **2** to Arg362 is bridged by a water molecule; the average distances from the phenolic oxygen atom to the carboxylate oxygen atom of Asp369 and the water oxygen atom are 2.9 Å and 2.3 Å, respectively; the average distance between the water oxygen atom and the closest guanidinium nitrogen atom of Arg362 is 3.3 Å.

### Synthesis

**Figure 3:**
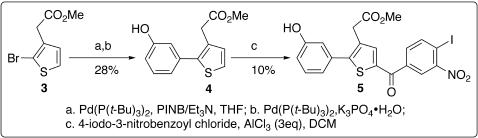
Synthetic scheme for intermediate 5.

**Figure 4:**
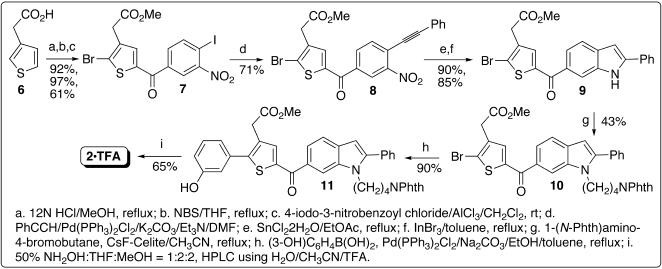
Synthetic scheme for inhibitor 2.

The initial synthesis of **2** followed a published scheme [7] that was devised to synthesize **1**. The starting material methyl 2-(2-(3-hydroxyphenyl)thiophen-3-yl)acetate (**4**) was prepared using Suzuki coupling [26]–[28] (Figure 3). However, the yield of Friedel-Crafts acylation [29], [30] for preparing **5** (Figure 3) was reduced to 10%, presumably owing to the hydroxyl group substituted at the phenyl ring. To increase the yield, a new scheme was devised to perform Friedel-Crafts acylation first and then Suzuki coupling (Figure 4); this scheme enables facile derivatization of the phenyl group substituted at the thiophene ring via a traditional or combinatorial chemistry approach. As shown in Figure 4, Heck alkynylation [31] of **7**, which carries both bromo and iodo atoms, was selectively achieved to afford **8** by using PhCCH/Pd(PPh_3_)_2_Cl_2_, K_2_CO_3_, and Et_3_N in DMF. A catalytic amount of InBr_3_ [32] was used for the indole formation to obtain **9** in a high yield. To obtain **10**, *N*-alkylation of the indole ring was carried out under the same conditions reported for the synthesis of **1** [7]. Refluxing **10** with 3-hydroxyphenylboronic acid in toluene:ethanol containing PdCl_2_(PPh_3_)_2_ and Na_2_CO_3_ gave **11** in a higher yield than a known procedure [33]. The final product **2** was obtained by a reaction of **11** with excess hydroxylamine [34] that simultaneously converted methyl ester and phthalimide to hydroxamic acid and amine, respectively [35], [36]. High-performance liquid chromatography (HPLC) purification using solvent containing trifluoroacetic acid yielded pure **2** in its trifluoroacetic acid salt form that was used for the following testing.

### Testing

**Figure 5:**
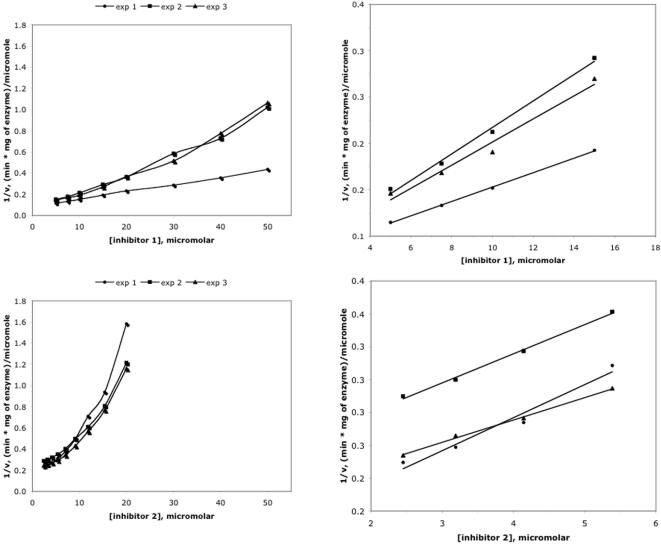
Dixon plots for inhibition of the botulinum neurotoxin serotype A endopeptidase by inhibitors 1 (upper panels) and 2 (lower panels). The right panels are the scale-up of the left panels.

HPLC-based kinetics assays [37] were used to measure the inhibition of BoNTA endopeptidase and a related endopeptidase from botulinum neurotoxin serotype B by **1** and **2**. A *K*_i_ value of 12±2.6 µM was previously obtained for **1** that was assumed to be a competitive inhibitor [7]. A further investigation on inhibitor effectiveness in this study revealed nonlinear Dixon plots for both **1** and **2** with data obtained at the condition of [S]<<*K*_M_ (Figure 5). The curvature of these plots indicates a partially competitive inhibition mechanism for **1** and **2** [38]; it precludes determination of *K*_i_ values using standard kinetic methods and invalidates the previously reported *K*_i_ value for **1**. To compare the relative inhibitory potencies of the two inhibitors, *K*_i_^app^ values of **1** and **2** were therefore obtained from the slope of 1/ν versus [I] at the conditions of [S]<<*K*_M_ and inhibitor concentrations less than 15.0 µM and 5.4 µM, respectively, according to a literature procedure [38], [39]. The Dixon plots for **1** and **2** are approximately linear at relatively low concentrations (Figure 5). Consequently, the kinetics assays showed that (1) at low inhibitor concentrations, **1** and **2** are competitive inhibitors of BoNTA endopeptidase with *K*_i_^app^ values of 7±2.4 µM and 3.8±0.8 µM, respectively, in HEPES buffer at pH 7.3, consistent with the half maximal inhibitory concentrations independently determined for the inhibition of BoNTA endopeptidase by **1** (15±1.5 µM) and **2 (**7.9±0.8 µM); (2) the *K*_i_^app^ values of **1** and **2** were unaffected after the zinc concentration of the assay was increased from 25 µM to 50 µM while holding all other experimental variables constant; (3) **1** and **2** did not inhibit botulinum neurotoxin serotype B endopeptidase at concentrations up to 20 µM (data not shown). These results preclude the possibility that the inhibition of BoNTA endopeptidase by **1** or **2** was due to nonspecific zinc chelation. All the observations demonstrate that **2** is a more potent BoNTA endopeptidase inhibitor than **1**.

## Discussion

### Improved Inhibitor of BoNTA Endopeptidase

Our results show that **1** and **2** are competitive inhibitors of BoNTA endopeptidase at inhibitor concentrations less than 15.0 µM and 5.4 µM, respectively, and that the *K*_i_^app^ values of **1** and **2** are 7±2.4 µM and 3.8±0.8 µM, respectively. However, both inhibitors are partially competitive inhibitors at relatively high inhibitor concentrations. These observations are not unprecedented. It has been reported that a Dixon plot of a dipeptide inhibitor (Gly-Trp) of angiotensin converting enzyme showed a competitive inhibition mechanism at relatively low inhibitor concentrations and a complex inhibition mechanism at relatively high concentrations, whereas the same inhibitor showed a competitive inhibition mechanism in a Lineweaver-Burk plot [39]. Interestingly, both BoNTA endopeptidase and angiotensin converting enzyme are zinc-bound enzymes. Further studies are needed to investigate the detailed inhibition mechanisms of **1** and **2** at relatively high concentrations. Nevertheless, the results of this study show clearly that **2** is a more potent BoNTA endopeptidase inhibitor than **1**, as evident from the Dixon plots shown in Figure 5 and from the half maximal inhibitory concentrations determined for the inhibition of BoNTA endopeptidase by **1** (15±1.5 µM) and **2** (7.9±0.8 µM).

### Efficiency of computer-aided optimization

While the optimization of **1** reported here was guided by MMDSs using the CaDA approach, 12 other analogs of **1** were made according to the scheme shown in Figure 4 using commercially available phenylboronic acids without the MMDSs guidance. Interestingly, none of these brute-force-approach-based derivatives was found to be more potent than **2** (data not shown). In terms of lead optimization for *in vitro* potency, which is an important step in drug development, the present work offers another demonstration of the relative efficiency of using computer simulations instead of the brute force approach for lead optimization. The results suggest that the CaDA approach is useful for both design and optimization of zinc protease inhibitors.

### Strategy for endopeptidase inhibitor optimization

As described above, the interface between BoNTA endopeptidase and its substrate is more than twice as large as typical protein-protein interfaces [16], [17]. This large interface causes a conflict in the design and optimization of medically useful BoNTA endopeptidase inhibitors in that, on the one hand, a large molecule is needed to gain more interactions at the unusually large interface, and on the other hand, a small molecule is required to ensure good cell permeability. To avoid the conflict, optimization should be directed towards improving inhibitor affinity with a small increase in molecular weight. This strategy is necessary in early stages of optimization of a micromolar lead because a bivalence or fragment-based approach will be used in later stages of the optimization to take advantage of peripheral sites or exosites on the endopeptidase to achieve high affinity [12], [13], [17], [40], [41]. The bivalence or fragment-based approach will be inapplicable to the optimization if the molecular weight of a lead is already too high. At present, the molecular weights of **1** and **2** are 524 and 540 Da, respectively. This study shows that it is practical to achieve a twofold improvement in inhibitory potency with a molecular weight increase of only 16 Da by adding one oxygen atom, given the guidance of the MMDSs of BoNTA endopeptidase using the CaDA approach. Further optimization of **2** following the example reported herein is underway and will be reported in due course.

## Materials and Methods

### Computational Methods

#### Preparation of the zinc- and inhibitor-bound endopeptidase

The initial structure of the zinc-containing BoNTA endopeptidase was taken from an available crystal structure of a BoNTA endopeptidase mutant (Glu224Gln and Tyr366Phe; Protein Data Bank code: 1XTG, residue numbers of 1XTG described in this section deviate by one from the residue numbers described in RESULTS and in Figure 2) [17]. To change the mutant back to the wildtype, residues 224 and 366 were mutated to Glu and Tyr, respectively, and the dihedral N-CA-CB-CG of Tyr366 was changed from 65.66 to –171.84° of arc. Hydrogen atoms of the endopeptidase were added by using the QUANTA97 program (Accelrys Software, Inc, San Diego, California). The zinc divalent cation in the crystal structure was replaced by the tetrahedron-shaped zinc divalent cation that has four cationic dummy atoms surrounding the central zinc ion [19] (for more information on zinc protein simulations see “http://mayoresearch.mayo.edu/mayo/research/camdl/zinc_protein.cfm”). His223, His227, and Glu262, which are the first-shell zinc coordinates, were treated as histidinate (HIN) and glutamate (GLU), respectively [19], [20], [42]–[44]. Glu261 and Glu351, which form a hydrogen bond with His223 and His227 respectively, were treated as glutamic acid (GLH) [19], [20], [42]–[44]. His170 and His269 were treated as the histidine whose epsilon nitrogen atom is protonated (HIE); His39 and His230 were treated as the histidine whose delta nitrogen atom is protonated (HID) and histidinium (HIP), respectively. Inhibitor **1** or **2** was manually docked into the active site of the endopeptidase according to a previously reported 3D model of inhibitor **1**-bound BoNTA endopeptidase [7]. One chloride ion was added next to the ammonium group of Lys381 located on the surface of the protein to neutralize the protein. The published force field parameters of the tetrahedron-shaped zinc divalent cation [7] were used in energy minimizations and in the following described molecular dynamics simulations. The RESP charges and the AMBER force field parameters of inhibitors **1** and **2** were generated by using the ANTECHAMBER module of the AMBER 7 program [45] and the structures of **1** and **2** that were optimized at the HF/6-31G* level with the Gaussian 98 Program [46] (Figure S1 and Tables S1 and S2). A 10,000-step energy minimization was first performed on inhibitor **2** with a positional constraint applied to the rest of the complex. A 50-step energy minimization was then performed on the tetrahedron-shaped zinc divalent cation and **2** with a positional constraint applied to the endopeptidase only. A 200-step energy minimization was lastly performed on the entire complex without any constraint.

#### Multiple molecular dynamics simulations

All MMDSs (2.0 ns for each simulation with a unique seed number for starting velocities of the system) were performed according to a published protocol [47] using the PMEMD module of the AMBER 8 program [45] with the Cornell et al. force field (parm99.dat) [48]. The topology and coordinate files used for the MMDSs were generated by the PREP, LINK, EDIT, and PARM modules of the AMBER 5 program [45]. All simulations used (1) a dielectric constant of 1.0; (2) the Berendsen coupling algorithm [49]; (3) a periodic boundary condition at a constant temperature of 300 K and a constant pressure of 1 atm with isotropic molecule-based scaling; (4) the Particle Mesh Ewald method to calculate long-range electrostatic interactions [50]; (5) iwrap = 1; (6) a time step of 1.0 fs; (7) the SHAKE-bond-length constraints applied to all the bonds involving the H atom; (8) default values of all other inputs of the PMEMD module. The initial structure of the **2**-bound BoNTA endopeptidase used for the MMDSs had no structural water molecules, and was solvated with TIP3P water molecules [25] (EDIT input: NCUBE = 10, QH = 0.4170, DISO = 2.20, DISH = 2.00, CUTX = 8.0, CUTY = 8.0, and CUTZ = 8.0). The solvated endopeptidase complex system was first energy-minimized for 200 steps to remove close van der Waals contacts in the solvated system, slowly heated to 300 K (10 K/ps), and then equilibrated for 1.5 ns. The CARNAL module was used for geometric analysis and for obtaining the time-average structure. All MMDSs were performed on 40 Apple G5 processors. The energy minimization of the time-average structure used “NCYC = 50” and other default inputs of the SANDER module of AMBER 5 [45], respectively.

#### Free energy perturbation calculation [24]

The absolute free energy of binding between **1** and BoNTA endopeptidase was obtained by perturbing **1** to nothing over 24 windows of perturbation using the thermodynamics integration method implemented in the SANDER module of AMBER 8 [45]. Each window was equilibrated and sampled with MMDSs (10 simulations) using the procedure described above. Each of the MMDSs used a 1.0-fs time step and lasted 0.5 ns and 1.0 ns for equilibration and sampling, respectively. The perturbation used 12/24-point Gaussian quadrature and the mixing rule shown in Equation 1. This exceptionally computing intensive calculation was performed on 480 Apple G5 processors for two months.
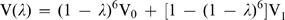
(Eq.1)


### Experimental Methods

#### Synthesis

The^ 1^H and ^13^C NMR spectra were recorded on a Varian Mercury 400 spectrometer. Chemical shifts are reported in ppm using the solvent resonance as the internal standard. Data are reported as follows: chemical shift, multiplicity (s = single, d = doublet, t = triplet, q = quartet, br = broad, and m = multiplet), integration, and coupling constants. High-resolution mass spectra were obtained on a Bruker BioTOF II ESI. THF and CH_2_Cl_2_ were dried using activated alumina columns from Solv-Tek (Berryville, VA). DMF and CH_3_CN were dried by distillation from CaH_2_ under N_2_. All other commercially obtained reagents were used as received. Medium-pressure liquid chromatography (MPLC) was performed with Biotage SP-1 (Charlottesville, VA) using silica gel 60 (EM Science, 230-400 mesh).

#### Methyl 2-(2-bromo-5-(4-iodo-3-nitrobenzoyl)thiophen-3-yl)acetate (7)

To a stirred solution of 2-(thiophen-3-yl)acetic acid (**6**, 28.00 g, 196.94 mmol) in methanol (300 mL) was added 12 N HCl (15 mL) and then refluxed for two hours. Methanol was removed by evaporation in vacuo, the residue was dissolved in dichloromethane, washed with saturated NaHCO_3_ solution, dried over MgSO_4_, filtered, and concentrated. Kugelrohr distillation of the crude product at 90°C per 0.1 mmHg gave the desired ester as a colorless oil (28.43 g, 92%).

^1^H NMR (400 MHz, CDCl_3_) δ 3.67 (s, 2H), 3.71 (s, 3H), 7.04 (dd, 1H, *J* = 1.2, 4.9 Hz), 7.20 (m, 1H), 7.29 (1H, dd, *J* = 2.0, 4.9 Hz).

To a solution of the above ester (10.00 g, 64.02 mmol) in THF (100 mL) was added NBS (11.40 g, 64.02 mmol). The resulting mixture was refluxed for two and a half hours. The solvent was removed in vacuo. MPLC purification (Hex:EtOAc/9:1) of the residue gave (2-bromothiophen-3-yl)acetic acid as a colorless oil (14.55 g, 97%).

^1^H NMR (400 MHz, CDCl_3_) δ 3.64 (s, 2H), 3.72 (s, 3H), 6.93 (d, 1H, *J* = 5.6 Hz), 7.25 (d, *J* = 5.6 Hz).

To a stirred solution of methyl (2-bromothiophen-3-yl)acetate (120 mg, 0.51 mmol) and 4-iodo-3-nitrobenzoyl chloride (150 mg, 0.48 mmol) in anhydrous CH_2_Cl_2_ (10 mL) was added AlCl_3_ (260 mg, 1.95 mmol) in four portions at 10-minute intervals at room temperature. The resulting mixture was stirred overnight. The reaction mixture was slowly poured onto 5 g of ice and allowed to warm to room temperature. The aqueous phase was extracted with CH_2_Cl_2_ (3×15 mL). The combined organic layer was dried over MgSO_4_, filtered, and then concentrated in vacuo. MPLC purification (Hex:EtOAc/5:1) of the residue gave **7** as a light yellow solid (158 mg, 61%).

^1^H NMR (400 MHz, CDCl_3_) δ 8.27 (d, 1H, *J = *2.0 Hz), 8.22 (d, 1H, *J = *8.0 Hz), 7.70 (dd, 1H, *J = *2.0, 8.2 Hz), 7.48 (s, 1H), 3.74 (s, 3H) and 3.68 (s, 2H);^ 13^C NMR (100 MHz, CDCl_3_) δ 183.8, 170.0, 153.2, 142.9, 141.9, 138.3, 136.5, 135.8, 133.1, 125.7, 124.5, 91.8, 52.6 and 35.0; HRMS-ESI calcd C_14_H_9_BrINO_5_S [M+Na] 531.8322, found 531.8331.

#### Methyl 2-(2-bromo-5-(3-nitro-4-(phenylethynyl)benzoyl)thiophen-3-yl)acetate (8)

A solution of **7** (150 mg, 0.29 mmol), phenylacetylene (32 µL, 0.29 mmol), Pd(PPh_3_)_2_Cl_2 _(21 mg, 0.03 mmol), K_2_CO_3_ (42 mg, 0.29 mmol), and Et_3_N (40 µL, 0.29 mmol) in DMF (3 mL) was stirred for 24 hours at room temperature. Water (5 mL) was added to the mixture and then extracted with EtOAc (3×10 mL). The combined organic layer was washed with brine, dried over MgSO_4_, and concentrated in vacuo. MPLC purification (Hex:EtOAc/5:1) of the residue gave **8** as a solid foam (101 mg, 71 %).

^1^H NMR (400 MHz, CDCl_3_) δ 8.54 (d, 1H, *J = *1.6 Hz,); 8.06 (dd, 1H, *J = *1.6, 8.0 Hz), 7.86 (d, 1H, *J = *8.4 Hz), 7.64 (m, 2H), 7.52 (s, 1H), 7.42 (m, 3H), 3.75 (s, 3H) and 3.70 (s, 2H); ^13^C NMR (100 MHz, CDCl_3_) δ 183.9, 170.1, 149.5, 142.2, 137.0, 136.3, 135.7, 135.3, 132.9, 132.5, 130.1, 128.8, 125.6, 124.3, 122.8, 122.1, 101.1, 84.7, 52.7 and 35.0; HRMS-ESI calcd C_22_H_14_BrNO_5_S [M+Na] 505.9668, found 505.9666.

#### Methyl (2-bromo-5-(2-phenyl-1*H*-indole-6-carbonyl)thiophen-3-yl)acetate (9)

To a solution of **8** (25 mg, 0.05 mmol) in EtOAc (5 mL) was added stannous chloride dihydrate (58 mg, 0.26 mmol). The resulting mixture was refluxed for one hour under N_2_. The reaction mixture was poured onto ice (5 g), and basified with saturated NaHCO_3_ solution to pH 8. The white milky mixture was filtered through a Celite pad to remove tin oxides. The organic layer from the filtrate was dried over MgSO_4_, filtered, and then concentrated in vacuo. MPLC purification (Hex:EtOAc/4:1) of the crude product gave the desired intermediate methyl (2-bromo-5-(3-amino-4-(phenylethynyl)benzoyl)thiophen-3-yl)acetate as a yellow foam (21 mg, 90%).

^1^H NMR (400 MHz, CDCl_3_) δ 7.55 (m, 2H), 7.50 (s, 1H), 7.48 (d, 1H, *J* = 1.6 Hz), 7.38 (m, 3H), 7.17 (m, 2H), 4.40 (br, 2H), 3.73 (s, 3H) and 3.66 (s, 2H); ^13^C NMR (100 MHz, CDCl_3_) δ 186.7, 170.3, 148.1, 143.4, 138.1, 135.9, 135.1, 132.4, 131.8, 129.0, 128.7, 122.9, 122.8, 118.8, 114.5, 112.3, 97.6, 85.3, 52.6 and 35.0; HRMS-ESI calcd C_22_H_16_BrNO_3_S [M+Na] 475.9926, found 475.9923.

To a 250 mL flask containing freshly distilled toluene (50 mL) were added methyl (2-bromo-5-(3-amino-4-(phenylethynyl)benzoyl)thiophen-3-yl)acetate (2.93 g, 6.45 mmol) and indium tribromide (1.14 g, 3.22 mmol) under N_2_. The resulting mixture was refluxed for one hour. The solvent was removed in vacuo. MPLC purification (Hex:EtOAc/4:1) of the crude product gave **9** as a yellow solid (2.50 g, 85%).

^1^H NMR (400 MHz, CDCl_3_) δ 9.38 (br, 1H), 7.98 (s, 1H), 7.69 (d, 2H, *J* = 7.8 Hz), 7.58 (m, 2H), 7.47 (s, 1H), 7.40 (t, 2H, *J = *7.5 Hz), 7.30 (m, 1 H), 6.80 (s, 1H), 3.65 (s, 3H) and 3.59 (s, 2H); ^13^C NMR (100 MHz, CDCl_3_) δ 187.4, 170.5, 144.2, 142.3, 136.5, 135.7, 134.9, 133.3, 131.8, 130.9, 129.4, 128.8, 125.9, 121.9, 120.4, 113.5, 100.3, 52.6 and 35.2; HRMS-ESI calcd C_22_H_16_BrNO_3_S [M+Na] 475.9926, found 475.9945.

#### Methyl 2-(2-bromo-5-(1-(4-(1,3-dioxoisoindolin-2-yl)butyl)-2-phenyl-1*H*-indole-6-carbonyl)-thiophene-3-yl)acetate (10)

To a stirred solution of **9** (45 mg, 0.10 mmol) in anhydrous CH_3_CN (5 mL) was added CsF-Celite (125 mg) under N_2_, followed by adding *N*-(4-bromobutyl)-phthalimide (28 mg, 0.10 mmol). The resulting mixture was refluxed for five hours, cooled to room temperature, and filtered. The filtrate was evaporated in vacuo. MPLC purification (Hex:EtOAc/4:1) of the residue gave **10** as a yellow solid (28 mg, 43%).

^1^H NMR (400 MHz, CDCl_3_) δ 7.98 (s, 1H), 7.74 (m, 6H), 7.59 (s, 1H), 7.47 (m, 5H), 6.60 (s, 1H), 4.32 (t, 2H, *J = *7.2 Hz), 3.73 (s, 3H), 3.71 (s, 2H), 3.52 (t, 2H, *J = *6.8 Hz), 1.72 (m, 2H) and 1.51 (m, 2H); ^13^C NMR (100 MHz, CDCl_3_) δ 187.3, 170.4, 168.5, 145.2, 144.3, 136.8, 135.6, 134.9, 134.2, 134.1, 132.5, 132.1, 130.7, 129.5, 129.0, 128.8, 123.5, 123.4, 121.5, 120.6, 112.5, 103.1, 52.5, 43.7, 37.3, 35.1, 27.4 and 25.8; HRMS-ESI calcd C_34_H_27_BrN_2_O_5_S [M+Na] 677.0716, found 677.0727.

#### Methyl 2-(5-(1-(4-(1,3-dioxoisoindolin-2-yl)butyl)-2-pheny-1*H*-indole-6-carbonyl-2-(3-**hydroxyphenyl)thiophen-3-yl)acetate (11)**

To a solution of 3-hydroxyphenylboronic acid (32 mg, 0.23 mmol) in EtOH (1 mL, degassed with N_2_), was added a solution of **10** (153 mg, 0.23 mmol) in toluene (5 mL), followed by adding Pd(PPh_3_)_2_Cl_2_ (16.3 mg, 0.023 mmol) and Na_2_CO_3 _(50 mg, 0.46 mmol). The reaction mixture was refluxed for three hours under N_2_, and then the solvent was removed in vacuo. MPLC purification (Hex:EtOAc/2:1) of the residue gave **11** as a yellow solid (140 mg, 90%).

^1^H NMR (400 MHz, CDCl_3_) δ 7.98 (s, 1H), 7.70 (m, 2H), 7.64–7.59 (m, 4H), 7.42–7.33 (m, 6H), 7.24 (t, 1H, *J = *7.9 Hz), 7.07 (t, 1H, *J* = 2.0 Hz), 6.97 (d, 1H, *J* = 7.6 Hz), 6.85 (dd, 1H, *J* = 2.0, 7.9 Hz), 6.52 (s, 1H), 4.23 (t, 2H, *J = *7.0 Hz), 3.67 (s, 3H), 3.62 (s, 2H), 3.45 (t, 2H, *J = *6.8 Hz), 1.67 (m, 2H) and 1.42 (m, 2H); ^13^C NMR (100 MHz, CDCl_3_) δ 188.6, 171.9, 168.8, 157.0, 149.2, 145.2, 141.6, 137.8, 136.7, 134.3, 132.6, 132.1, 131.3, 130.5, 130.4, 129.5, 129.0, 128.8, 123.5, 121.7, 121.3, 120.7, 116.5, 112.8, 103.2, 52.6, 43.7, 37.5, 34.6, 27.5 and 25.9; HRMS-ESI calcd C_40_H_32_N_2_O_6_S [M+Na] 691.1873, found 691.1892.

#### 2-(5-1-(4-Aminobutyl)-2-phenyl-1*H*-indole-6-carbonyl)-2-(3-hydroxyphenyl)-thiophen-3-yl)-*N*-hydroxyacetamide trifluoroacetic acid salt (2•TFA)

To a stirred solution of **11** (50 mg, 0.07 mmol) in THF/MeOH (1 mL each), 1 mL of 50% aqueous NH_2_OH was added, followed by adding a catalytic amount of KCN (CAUTION: KCN is highly toxic and must be handled with extreme care by trained personnel). The resulting mixture was stirred overnight at room temperature. After the solvent was removed in vacuo, the residue was washed with water (3×5 mL). HPLC purification of the residue gave **2•TFA** as a yellow powder (32 mg, 65%). HPLC purification condition: Phenomenex Gemini 5 µm, C18, 4.6×250 mm, eluting with linear gradient of 20% of solution A (1000 mL of H_2_O and 45 µL of TFA) to 100% of solution B (100 mL of H_2_O, 900 mL of CH_3_CN, and 45 µL of TFA) over 20 minutes, and flow rate of 1.5 mL/min with a retention time of 12.20 minutes for **2•TFA** (see Figure S2 for chromatograms of **2** before and after the HPLC purification).

^1^H NMR (400 MHz, DMSO-*d*_6_) δ 10.82 (s, 1H), 9.82 (br, 1H), 9.01 (s, 1H), 8.11 (s, 1H), 7.80 (d, 1H, *J = *2.1 Hz), 7.74 (m, 1H), 7.59–7.50 (m, 6H), 7.30 (m, 1H), 7.04 (d, 1H, *J = *8.0 Hz), 6.99 (s, 1H), 6.87 (d, 1H, *J = *8.2 Hz), 6.70 (s, 1H), 4.34 (m, 2H), 3.43 (s, 2H), 2.63 (m, 2H), 1.65 (m, 2H) and 1.31 (m, 2H); ^13^C NMR (100 MHz, DMSO-*d*_6_) δ 187.7, 167.4, 158.5, 148.2, 145.2, 141.5, 138.4, 136.9, 134.3, 133.0, 132.5, 131.7, 131.2, 130.9, 129.8, 129.6, 129.4, 121.2, 121.0, 120.4, 116.7, 116.4, 113.2, 103.3, 43.8, 39.1, 33.1, 27.4 and 25.0; DEPT-135 ^13^C NMR (100 MHz, CD_3_OD) δ 137.6 (CH), 130.9 (CH), 130.1 (CH), 129.3 (CH), 128.8 (CH), 128.7 (CH), 120.7 (CH), 120.6 (CH), 120.3 (CH), 115.9 (CH), 113.0 (CH), 102.9 (CH), 43.4 (CH_2_), 39.2 (CH_2_), 32.3 (CH_2_), 27.1 (CH_2_) and 24.7 (CH_2_); HRMS-ESI calcd C_31_H_29_N_3_O_4_S [M+H] 540.1942, found 540.1934.

#### Botulinum neurotoxin inhibition assays

Assays of protease activities of BoNTA and botulinum neurotoxin serotype B (BoNTB) were done at 37°C and contained 0.5 mM substrate, 0.5–1.5 µg/mL recombinant BoNTA/BoNTB endopeptidase, 40 mM HEPES, and 0.05% tween at pH 7.3. BoNTA endopeptidase assays also contained 1 mM dithiothreitol, 25 µM ZnCl_2_, and 0.5 mg/mL bovine serum albumin, while BoNTB endopeptidase assays were supplemented with 1 mM dithiothreitol only. Substrate for BoNTA was a peptide containing residues 187–203 of SNAP-25 [37], while that for BoNTB was residues 60–94 of VAMP [51]. Inhibitors were dissolved in dimethyl sulfoxide at 10 times the final assay concentration, then diluted into the assay mixture containing substrate, followed by addition of endopeptidase (i.e., inhibitor and endopeptidase were not preincubated). Assay times and endopeptidase concentrations were adjusted so that less than 10% of the substrate was hydrolyzed. Assays were stopped by acidification with trifluoroacetic acid and analyzed by reverse-phase HPLC as described [37]. Inhibition of BoNTA endopeptidase activity by **1** or **2** was determined in three independent experiments using eight and nine concentrations of **1** and **2** in each, respectively.

#### Determination of *K*_i_^app^

For both inhibitors, Dixon plots were nonlinear when the entire range of tested concentrations was included, suggesting a partially competitive inhibition mechanism. Nonetheless, replots of the linear portion of the data, where [S]<<*K*_M_ and inhibitor concentrations of **1** and **2** were less than 15.0 µM and 5.4 µM respectively, permitted calculations of *K*_i_^app^ values for **1** and **2** using the previously established *K*_M_ for the substrate measured in the absence of inhibitor [52] according to a literature procedure [38], [39].

## Supporting Information

Table S1 The AMBER atom types and charges of inhibitors of 1 and 2.
(0.15 MB DOC)

Table S2 The AMBER force field parameters of inhibitors of 1 and 2.
(0.12 MB DOC)

Figure S1 Definition of atom names of inhibitors of 1 and 2.
(9.48 MB TIF)

Figure S2 Chromatograms of inhibitor 2 before and after the HPLC purification.
(1.33 MB TIF)

## References

[1] Shapiro RL, Hatheway C, Swerdlow DL (1998). Botulism in the United States-a clinical and epidemiologic review.. Ann Intern Med.

[2] Kessler KR, Benecke R (1997). Botulinum toxin—from poison to remedy.. Neurotoxicology.

[3] Springen K, Raymond J, Skipp C, Scelfo JSS (2002). The Botox boom.. Newsweek.

[4] Singh BR (2000). Intimate details of the most poisonous poison.. Nat Struct Biol.

[5] Simpson LL (1981). The origin, structure, and pharmacological activity of botulinum toxin.. Pharmacol Rev.

[6] Burnett JC, Schmidt JJ, Stafford RG, Panchal RG, Nguyen TL (2003). Novel small molecule inhibitors of botulinum neurotoxin A metalloprotease activity.. Biochem Biophys Res Commun.

[7] Park JG, Sill PC, Makiyi EF, Garcia-Sosa AT, Millard CB (2006). Serotype-selective, small-molecule inhibitors of the zinc endopeptidase of botulinum neurotoxin serotype A. Bioorg Med Chem.

[8] Boldt GE, Eubanks LM, Janda KD (2006). Identification of a botulinum neurotoxin A protease inhibitor displaying efficacy in a cellular model.. Chem Commun (Camb).

[9] Boldt GE, Kennedy JP, Hixon MS, McAllister LA, Barbieri JT (2006). Synthesis, characterization and development of a high-throughput methodology for the discovery of botulinum neurotoxin a inhibitors.. J Comb Chem.

[10] Boldt GE, Kennedy JP, Janda KD (2006). Identification of a potent botulinum neurotoxin a protease inhibitor using in situ lead identification chemistry.. Org Lett.

[11] Dickerson TJ, Janda KD (2006). The use of small molecules to investigate molecular mechanisms and therapeutic targets for treatment of botulinum neurotoxin A intoxication.. Chem Biol.

[12] Merino I, Thompson JD, Millard CB, Schmidt JJ, Pang Y-P (2006). Bis-imidazoles as molecular probes for peripheral sites of the zinc endopeptidase of botulinum neurotoxin serotype A.. Bioorg Med Chem.

[13] Johnson SL, Pellecchia M (2006). Structure- and fragment-based approaches to protease inhibition.. Curr Top Med Chem.

[14] Eubanks LM, Hixon MS, Jin W, Hong S, Clancy CM (2007). An in vitro and in vivo disconnect uncovered through high-throughput identification of botulinum neurotoxin A antagonists.. Proc Natl Acad Sci U S A.

[15] Burnett JC, Ruthel G, Stegmann CM, Panchal RG, Nguyen TL (2007). Inhibition of metalloprotease botulinum serotype A from a pseudo-peptide binding mode to a small molecule that is active in primary neurons.. J Biol Chem.

[16] Arkin MR, Wells JA (2004). Small-molecule inhibitors of protein-protein interactions: progressing towards the dream.. Nat Rev Drug Dis.

[17] Breidenbach MA, Brunger AT (2004). Substrate recognition strategy for botulinum neurotoxin serotype A.. Nature.

[18] Roe RR, Pang Y-P (1999). Zinc's exclusive tetrahedral coordination governed by its electronic structure.. J Mol Model.

[19] Pang YP (1999). Novel zinc protein molecular dynamics simulations: steps toward antiangiogenesis for cancer treatment.. J Mol Model.

[20] Pang YP, Xu K, El Yazal J, Prendergast FG (2000). Successful molecular dynamics simulation of the zinc-bound farnesyltransferase using the cationic dummy atom approach.. Protein Sci.

[21] Pang Y-P (2001). Successful molecular dynamics simulation of two zinc complexes bridged by a hydroxide in phosphotriesterase using the cationic dummy atom method.. Proteins.

[22] Oelschlaeger P, Schmid RD, Pleiss J (2003). Insight into the mechanism of the IMP-1 metallo-beta-lactamase by molecular dynamics simulations.. Protein Eng.

[23] Oelschlaeger P, Schmid RD, Pleiss J (2003). Modeling domino effects in enzymes: molecular basis of the substrate specificity of the bacterial metallo-beta-lactamases IMP-1 and IMP-6.. Biochemistry.

[24] Pang YP, Miller JL, Kollman PA (1999). Computational and experimental studies of (2,2)-bis(indol-1-yl-methyl)acetate suggest the importance of the hydrophobic effect in aromatic stacking interactions.. J Am Chem Soc.

[25] Jorgensen WL, Chandreskhar J, Madura JD, Impey RW, Klein ML (1982). Comparison of simple potential functions for simulating liquid water.. J Chem Phys.

[26] Miyaura N, Suzuki A (1979). Stereoselective synthesis of arylated (E)-alkenes by the reaction of alk-1-enylboranes with aryl halides in the presence of palladium catalyst.. J Chem Soc, Chem Commun.

[27] Suzuki A (1991). Synthetic studies via the cross-coupling reaction of organoboron derivatives with organic halides.. Pure Appl Chem.

[28] Miyaura N, Suzuki A (1995). Palladium-catalyzed cross-coupling reactions of organoboron compounds.. Chem Rev.

[29] Joule JA, Mills K, Smith GF (1998). Heterocyclic Chemistry..

[30] Gupta RR, Kumar M, Gupta V (1999). Heterocyclic Chemistry. Volume II..

[31] Dieck HA, Heck FR (1975). Palladium catalyzed synthesis of aryl heterocyclic and vinylic acetylene derivatives.. J Organomet Chem.

[32] Sakai N, Annaka K, Konakahara T (2004). Palladium-catalyzed coupling reaction of terminal alkynes with aryl iodides in the presence of indium tribromide and its application to a one-pot synthesis of 2-phenylindole.. Org Lett.

[33] Meegalla SK, Doller D, Sha D, Soll R, Wisnewski N (2004). Synthesis and GABA receptor potency of 3-thiomethyl-4-(hetero)aryl-5-amino-1-phenylpyrazoles.. Bioorg Med Chem Lett.

[34] Ho CY, Strobel E, Ralbovsky J, Galemmo RA (2005). Improved solution- and solid-phase preparation of hydroxamic acids from esters.. J Org Chem.

[35] Mootoo DR, Fraiser-Reid B (1989). n-Pentenyl 2-amino-2-deoxy glycosides undergo stereoselective coupling under mild, chemospecific conditions.. Tetrahedron Lett.

[36] Ariffin A, Khan MN, Lan LC, May FY, Yun CS (2004). Suggested improved method for the Ing-Manske and related reactions for the second step of Gabriel synthesis of primary amines.. Synth Commun.

[37] Schmidt JJ, Bostian KA (1997). Endoproteinase activity of type A botulinum neurotoxin: substrate requirements and activation by serum albumin.. J Protein Chem.

[38] Todhunter JA (1979). Reversible enzyme inhibition.. Methods in Enzymology.

[39] Shapiro R, Riordan JF (1984). Inhibition of angiotensin converting enzyme: mechanism and substrate dependence.. Biochemistry.

[40] Pang Y-P, Quiram P, Jelacic T, Hong F, Brimijoin S (1996). Highly potent, selective, and low cost bis-tetrahydroaminacrine inhibitors of acetylcholinesterase: steps toward novel drugs for treating Alzheimer's disease.. J Biol Chem.

[41] Pang Y-P (2004). Nonbonded bivalence approach to cell-permeable molecules that target DNA sequences.. Bioorg Med Chem.

[42] El Yazal J, Pang YP (1999). *Ab initio* calculations of proton dissociation energies of zinc ligands: hypothesis of imidazolate as zinc ligand in proteins.. J Phys Chem B.

[43] El Yazal J, Roe RR, Pang Y-P (2000). Zinc's affect on proton transfer between imidazole and acetate predicted by ab initio calculations.. J Phys Chem B.

[44] El Yazal J, Pang YP (2001). Comparison of DFT, Moller-Plesset, and coupled cluster calculations of the proton dissociation energies of imidazole and N-methylacetamide in the presence of zinc(II).. J Mol Struct (Theochem),.

[45] Pearlman DA, Case DA, Caldwell JW, Ross WS, Cheatham TE (1995). AMBER, a package of computer programs for applying molecular mechanics, normal mode analysis, molecular dynamics and free energy calculations to simulate the structural and energetic properties of molecules.. Comput Phys Commun.

[46] Frisch MJ, Trucks GW, Schlegel HB, Gill PMW, Hohnson BG (1999). GAUSSIAN 98, Revision A.7..

[47] Pang Y-P (2004). Three-dimensional model of a substrate-bound SARS chymotrypsin-like cysteine proteinase predicted by multiple molecular dynamics simulations: catalytic efficiency regulated by substrate binding.. Proteins.

[48] Cornell WD, Cieplak P, Bayly CI, Gould IR, Merz KM (1995). A second generation force field for the simulation of proteins, nucleic acids, and organic molecules.. J Am Chem Soc.

[49] Berendsen HJC, Postma JPM, van Gunsteren WF, Di Nola A, Haak JR (1984). Molecular dynamics with coupling to an external bath.. J Chem Phys.

[50] Darden TA, York DM, Pedersen LG (1993). Particle Mesh Ewald: An N log(N) method for Ewald sums in large systems.. J Chem Phys.

[51] Shone CC, Roberts AK (1994). Peptide substrate specificity and properties of the zinc-endopeptidase activity of botulinum type B neurotoxin.. Eur J Biochem.

[52] Schmidt JJ, Stafford RG (2003). Fluorigenic substrates for the protease activities of botulinum neurotoxins, serotypes A, B, and F.. Appl Environ Microbiol.

