# Supplementary material for: Computer-Aided Lead Optimization: Improved Small-Molecule Inhibitor of the Zinc Endopeptidase of Botulinum Neurotoxin Serotype A
Source: PLoS One. 2007 Aug 22;2(8):e761. doi: 10.1371/journal.pone.0000761 (PMC1942119; doi:10.1371/journal.pone.0000761)
Supplement: Table S1 — The AMBER atom types and charges of inhibitors of 1 and 2. (0.15 MB DOC) [file pone.0000761.s001.doc]

**Table S1. The AMBER atom types and charges of inhibitors of 1 and 2.**

| Inhibitor **1** | | | Inhibitor **2** | | |
| --- | --- | --- | --- | --- | --- |
| Atom name* | Atom type | Charge | Atom name | Atom type | Charge |
| C1 | CA | -0.096 | C1 | CA | -0.011 |
| H1 | HA | 0.15 | H1 | HA | 0.109 |
| C2 | CA | -0.208 | C2 | CA | -0.304 |
| H2 | HA | 0.149 | H2 | HA | 0.182 |
| C3 | CA | -0.068 | C3 | CA | -0.125 |
| H3 | HA | 0.132 | H3 | HA | 0.16 |
| C4 | CA | -0.208 | C4 | CA | 0.275 |
| H4 | HA | 0.149 | O1 | OH | -0.604 |
| C5 | CA | -0.096 | H4 | HO | 0.443 |
| H5 | HA | 0.15 | C5 | CA | -0.187 |
| C6 | CA | 0.069 | H5 | HA | 0.139 |
| C7 | C* | -0.075 | C6 | CA | 0.005 |
| C8 | C* | -0.038 | C7 | C* | -0.022 |
| C9 | CT | -0.08 | C8 | C* | 0.017 |
| C10 | C | 0.423 | C9 | CT | -0.085 |
| O1 | O | -0.564 | C10 | C | 0.454 |
| N1 | N | -0.134 | O2 | O | -0.614 |
| H6 | H | 0.12 | N1 | N | -0.192 |
| O2 | O | -0.724 | H6 | H | 0.24 |
| H8 | HC | 0.064 | O3 | O | -0.694 |
| H9 | HC | 0.064 | H7 | HC | 0.041 |
| C11 | C* | -0.115 | H8 | HC | 0.041 |
| H10 | HA | 0.147 | C11 | C* | -0.089 |
| S1 | S | -0.096 | H9 | HA | 0.188 |
| C12 | C* | -0.075 | S1 | S | -0.117 |
| C13 | C | 0.547 | C12 | C* | -0.094 |
| O3 | O | -0.481 | C13 | C | 0.516 |
| C14 | CA | -0.092 | O4 | O | -0.557 |
| C16 | CA | -0.196 | C14 | CA | -0.087 |
| H12 | HA | 0.153 | C16 | CA | -0.053 |
| C17 | CA | -0.168 | H11 | HA | 0.127 |
| H13 | HA | 0.158 | C17 | CA | -0.222 |
| C15 | CA | -0.175 | H12 | HA | 0.211 |
| H11 | HA | 0.216 | C15 | CA | -0.26 |
| C20 | CB | 0.025 | H10 | HA | 0.175 |
| C18 | CB | 0.115 | C20 | CB | 0.057 |
| C19 | C* | -0.394 | C18 | CB | 0.157 |
| H14 | HA | 0.191 | C19 | C* | -0.392 |
| C25 | C* | 0.021 | H13 | HA | 0.195 |
| C26 | CA | 0.076 | C25 | C* | 0.071 |
| C27 | CA | -0.08 | C26 | CA | 0.044 |
| H25 | HA | 0.117 | C27 | CA | -0.067 |
| C28 | CA | -0.207 | H24 | HA | 0.11 |
| H26 | HA | 0.157 | C28 | CA | -0.205 |
| C29 | CA | -0.098 | H25 | HA | 0.156 |
| H27 | HA | 0.138 | C29 | CA | -0.094 |
| C30 | CA | -0.207 | H26 | HA | 0.139 |
| H28 | HA | 0.157 | C30 | CA | -0.205 |
| C31 | CA | -0.08 | H27 | HA | 0.156 |
| H29 | HA | 0.117 | C31 | CA | -0.067 |
| N2 | N* | 0.021 | H28 | HA | 0.11 |
| C21 | CT | -0.091 | N2 | N* | -0.105 |
| H15 | H1 | 0.1 | C21 | CT | -0.035 |
| H16 | H1 | 0.1 | H14 | H1 | 0.071 |
| C22 | CT | -0.084 | H15 | H1 | 0.071 |
| H17 | HC | 0.042 | C22 | CT | -0.012 |
| H18 | HC | 0.042 | H16 | HC | 0.026 |
| C23 | CT | 0.053 | H17 | HC | 0.026 |
| H19 | HC | 0.039 | C23 | CT | -0.004 |
| H20 | HC | 0.039 | H18 | HC | 0.03 |
| C24 | CT | -0.04 | H19 | HC | 0.03 |
| H21 | HP | 0.038 | C24 | CT | 0.04 |
| H22 | HP | 0.038 | H20 | HP | 0.07 |
| N3 | N3 | 0 | H21 | HP | 0.07 |
| H7 | H | 0.218 | N3 | N3 | -0.384 |
| H23 | H | 0.218 | H22 | H | 0.312 |
| H24 | H | 0.218 | H23 | H | 0.312 |
|  |  |  | H29 | H | 0.312 |

*The atom names are defined in Figure S1.
