# Supplementary material for: Computer-Aided Lead Optimization: Improved Small-Molecule Inhibitor of the Zinc Endopeptidase of Botulinum Neurotoxin Serotype A
Source: PLoS One. 2007 Aug 22;2(8):e761. doi: 10.1371/journal.pone.0000761 (PMC1942119; doi:10.1371/journal.pone.0000761)
Supplement: Table S2 — The AMBER force field parameters of inhibitors of 1 and 2. (0.12 MB DOC) [file pone.0000761.s002.doc]

**Table S2**. The AMBER force field parameters of inhibitors of **1** and **2**.

| bond | K [ kcal/mol Å^2^)] | | | | R_eq_ (Å) | | |
| --- | --- | --- | --- | --- | --- | --- | --- |
| CA-C* | 473.7 | | | | 1.39 | | |
| C*-C* | 473.7 | | | | 1.39 | | |
| C*-S | 307.7 | | | | 1.7 | | |
| C*-C | 346.5 | | | | 1.49 | | |
| C*-N* | 440.2 | | | | 1.37 | | |
| angle | K [kcal/mol radian^2^)] | | | | T_eq_ (deg.) | | |
| CA-CA-C* | 63 | | | | 120 | | |
| CA-C*-C* | 63 | | | | 120 | | |
| CA-C*-S | 61.1 | | | | 120.79 | | |
| C*-C*-CT | 61.4 | | | | 122.59 | | |
| C*-C*-C* | 63 | | | | 120 | | |
| C*-S -C* | 66.5 | | | | 95.23 | | |
| C*-C*-S | 61.1 | | | | 120.79 | | |
| C*-CT-C | 64.161 | | | | 110.965 | | |
| C*-C*-HA | 50 | | | | 120 | | |
| C*-C*-C | 62 | | | | 122.13 | | |
| S -C*-C | 73.986 | | | | 117.575 | | |
| C*-C -O | 67.1 | | | | 126.4 | | |
| C*-C -CA | 64.1 | | | | 114.03 | | |
| O -C -CA | 67.1 | | | | 126.4 | | |
| CA-CB-N* | 68.7 | | | | 111.2 | | |
| CB-CB-C* | 63 | | | | 120 | | |
| CB-N*-C* | 67.7 | | | | 110.55 | | |
| CB-C*-HA | 50 | | | | 120 | | |
| CB-C*-C* | 63 | | | | 120 | | |
| C*-C*-N* | 68.7 | | | | 111.2 | | |
| C*-N*-CT | 62.7 | | | | 123.21 | | |
| CA-C*-N* | 68.7 | | | | 111.2 | | |
| dihedral | IDIVF | V_n_/2 (kcal/mol) | | γ (deg.) | | N | |
| CA-CA-C*-C* | 1 | 3.625 | | 180 | | 2 | |
| CA-CA-C*-S | 1 | 3.625 | | 180 | | 2 | |
| CA-C*-C*-CT | 1 | 3.625 | | 180 | | 2 | |
| CA-C*-C*-C* | 1 | 3.625 | | 180 | | 2 | |
| CA-C*-S -C* | 1 | 0.4 | | 180 | | 2 | |
| C*-C*-C*-HA | 1 | 3.625 | | 180 | | 2 | |
| C*-C*-C*-C* | 1 | 3.625 | | 180 | | 2 | |
| C*-S -C*-C* | 1 | 0.4 | | 180 | | 2 | |
| C*-S -C*-C | 1 | 0.4 | | 180 | | 2 | |
| C*-C*-C*-S | 1 | 3.625 | | 180 | | 2 | |
| C*-C*-C*-C | 1 | 3.625 | | 180 | | 2 | |
| CT-C*-C*-S | 1 | 3.625 | | 180 | | 2 | |
| CT-C*-C*-HA | 1 | 3.625 | | 180 | | 2 | |
| CT-C*-C*-C* | 1 | 3.625 | | 180 | | 2 | |
| C*-C*-C -O | 1 | 3.625 | | 180 | | 2 | |
| C*-C*-C -CA | 1 | 3.625 | | 180 | | 2 | |
| HA-C*-C*-S | 1 | 3.625 | | 180 | | 2 | |
| HA-C*-C*-C | 1 | 3.625 | | 180 | | 2 | |
| S -C*-C -O | 1 | 3.625 | | 180 | | 2 | |
| S -C*-C -CA | 1 | 3.625 | | 180 | | 2 | |
| CB-N*-C*-C* | 1 | 0.3 | | 180 | | 2 | |
| CB-N*-C*-CA | 1 | 0.3 | | 180 | | 2 | |
| CB-C*-C*-CA | 1 | 3.625 | | 180 | | 2 | |
| CB-C*-C*-N* | 1 | 3.625 | | 180 | | 2 | |
| C*-C*-N*-CT | 1 | 0.3 | | 180 | | 2 | |
| HA-C*-C*-CA | 1 | 3.625 | | 180 | | 2 | |
| HA-C*-C*-N* | 1 | 3.625 | | 180 | | 2 | |
| CA-C*-N*-CT | 1 | 0.3 | | 180 | | 2 | |
| CA-CA-C*-N* | 1 | 3.625 | | 180 | | 2 | |
| Improper dihedral | | | V_n_/2 (kcal/mol) | | γ (deg.) | | N |
| C*-CA-C*-S | | | 1.1 | | 180 | | 2 |
| C*-C*-C*-HA | | | 1.1 | | 180 | | 2 |
| C*-C -C*-S | | | 1.1 | | 180 | | 2 |
| CA-CB-CB-N* | | | 1.1 | | 180 | | 2 |
| C*-CA-CB-CB | | | 1.1 | | 180 | | 2 |
| C*-CB-C*-HA | | | 1.1 | | 180 | | 2 |
| C*-CA-C*-N* | | | 1.1 | | 180 | | 2 |
