# Supplementary figures and images for: Computer-Aided Lead Optimization: Improved Small-Molecule Inhibitor of the Zinc Endopeptidase of Botulinum Neurotoxin Serotype A
Source: PLoS One. 2007 Aug 22;2(8):e761. doi: 10.1371/journal.pone.0000761 (PMC1942119; doi:10.1371/journal.pone.0000761)

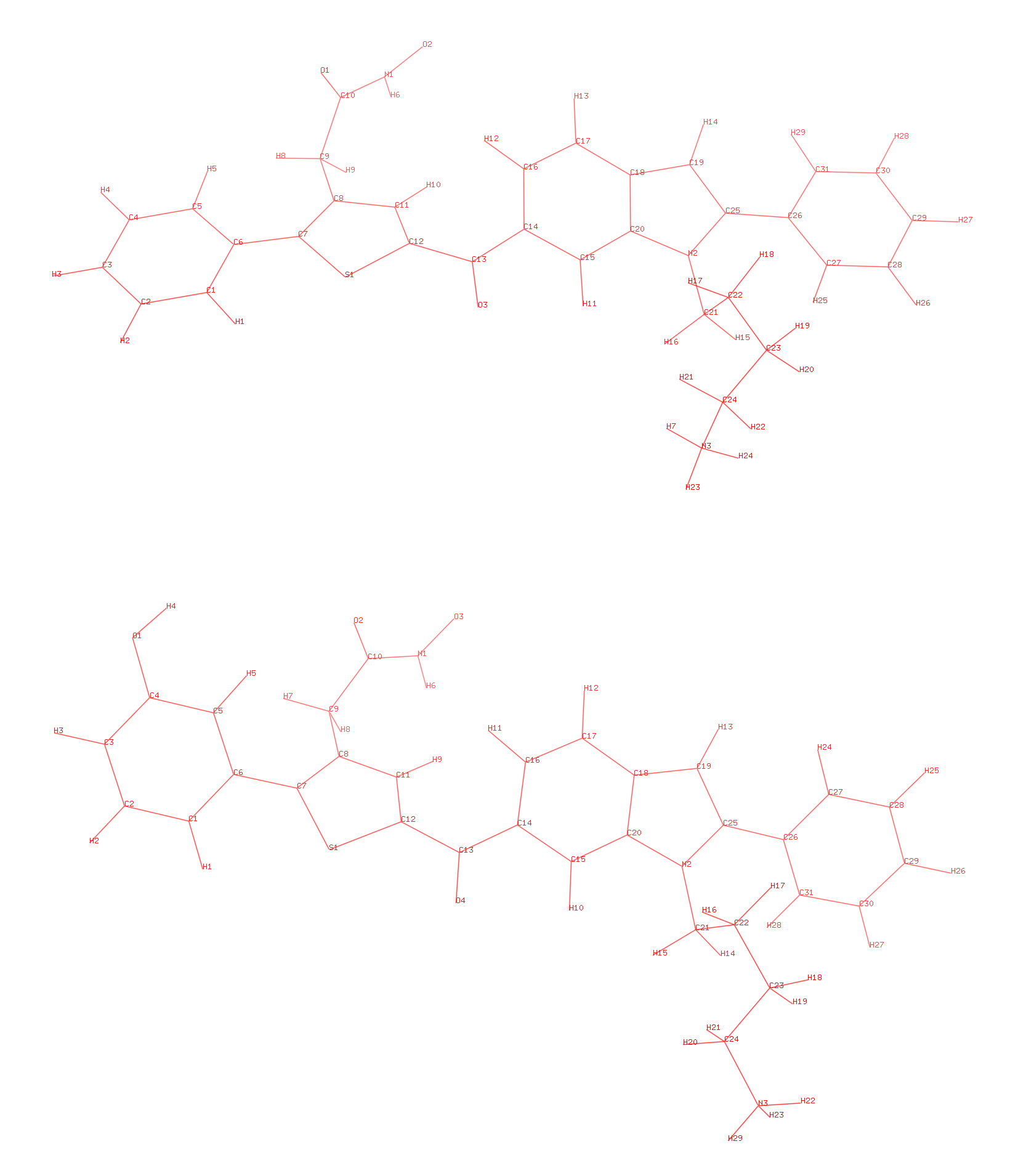

Supplement: Figure S1 — Definition of atom names of inhibitors of 1 and 2. (9.48 MB TIF) [file pone.0000761.s003.tif]
